# Predictive Values of PET/CT in Combination With Regulatory B Cells for Therapeutic Response and Survival in Contemporary Patients With Newly Diagnosed Multiple Myeloma

**DOI:** 10.3389/fimmu.2021.671904

**Published:** 2021-08-19

**Authors:** Jian Cui, Zhongqing Zou, Jiayu Duan, Wenjiao Tang, Yan Li, Li Zhang, Ling Pan, Ting Niu

**Affiliations:** ^1^Department of Hematology, West China Hospital, Sichuan University, Chengdu, China; ^2^West China School of Medicine, Sichuan University, Chengdu, China; ^3^Department of Hematology, Clinical Medical College & Affiliated Hospital of Chengdu University, Chengdu University, Chengdu, China

**Keywords:** myeloma, PET/CT, regulatory B cells, prognosis, tumor microenvironment

## Abstract

To assess patients with multiple myeloma (MM), the whole-body positron-emission tomography/computed tomography (PET/CT) occupies a pivotal position for diagnostic stratification, response evaluation, and survival prediction, while important limitations are recognized as incapable of representing tumor microenvironment. Regulatory B cells (Bregs) have been reported to have an inhibitory immune function, contributing to bone marrow (BM)-immunosuppressive microenvironment for MM. Therefore, to investigate the role of PET/CT in combination with Bregs’ ratios to predict therapeutic response and survival, we sequentially enrolled 120 patients with newly diagnosed MM (NDMM) who were treated with novel agents in our center, while conventional PET/CT parameters including maximum standard uptake value (SUVmax), ratios of BM-derived Bregs within CD19^+^ B cells, and patients’ clinical characteristics were collected. After a median follow-up of 28.20 months (range 7.00–46.93 months), SUVmax > 4.2 at onset, accounting for 53.2% of NDMM, was uncovered to predict inferior progression-free survival (PFS) as well as overall survival (OS). With regard to the ratios of BM-derived Bregs within CD19^+^ B cells, the cohort with the Bregs’ proportions lower than 10%, accounting for 46.2%, exerted poorer OS. Additionally, the patients with both SUVmax > 4.2 and Bregs’ ratios < 10%, accounting for 31.7%, yielded compromised therapeutic response and long-term survival. Collectively, this study may draw attention on the prognostic value of combination of PET/CT and Bregs’ ratios when clinical decisions are made for MM in the era of novel agents.

## Introduction

Risk stratifications of newly diagnosed multiple myeloma (NDMM) have been assessed by a number of prognostic variables. The International Staging System (ISS) and the revised ISS (R-ISS), which combine biochemical indicators and cytogenetic abnormalities (1, 2), are the most widely used. However, neither ISS nor R-ISS can reflect tumor biology determined by bone destruction and abnormalities of tumor microenvironment (TME), which are important pathogenic factors in MM (3, 4). Additionally, the reliability of cytogenetic results is unavoidably influenced by the uncertainty of collecting clonal plasma cells due to local tumor infiltration in MM’s bone marrow (BM).

Positron-emission tomography/computed tomography (PET/CT) with fluorine-18 fluorodeoxyglucose (^18^F-FDG) has been applied for the assessment of systemic tumor burden, response evaluation, and survival prediction in MM (5–9), for it provides ‘functional’ information regarding lesions as well as metabolic and anatomic information. Compared with traditional X-rays, systemic CT is a sensitive technique to detect the presence of bone lesions and/or BM involvement at the onset of MM (7). Moreover, ^18^F-FDG uptake in bone lesions represents the tumor metabolic activity, and maximum standard uptake value (SUVmax) is considered to have prognostic value in NDMM (6, 10–12).

In addition to systemic tumor burden, the BM immune TME, including T cells (13), osteoclasts (14) and extracellular matrix (15), are also involved in the occurrence, disease progression, and drug resistance of MM. Regulatory B cells (Bregs) are currently uncovered to be immunosuppressive cells that exist in the BM immune TME of MM (16). In previous studies, we first characterized CD19^+^CD24^hi^CD38^hi^ Bregs in BM samples from MM patients by flow cytometry (FCM) (17–19). We also explained the relationship between Bregs and disease status in MM that ratios of BM-derived Bregs within CD19^+^ B cells were significantly higher in patients with NDMM than in those on maintenance therapy after response (17). Bregs were also reported to predict progression-free survival (PFS) for patients with relapsed or refractory MM (20). However, prognostic impact of Bregs for NDMM patients has been still unclear.

To further uncover prognostic roles of TME-derived Bregs and systemic PET/CT, in this study, we herein report the results of a retrospective analysis collecting PET/CT scan and Bregs detection performed at baseline for patients with NDMM who received bortezomib plus dexamethasone-based (BD) therapy in southwest China.

## Methods

### Patients and Treatment Protocols

NDMM patients were sequentially enrolled from April 2017 to July 2020 at West China Hospital if they were over the age of 18 years, received PET/CT scan or FCM detection of Bregs at the onset of disease, and newly diagnosed with symptomatic MM based on the International Myeloma Working Group (IMWG) (21) diagnostic criteria. The final follow-up ended in May 31, 2021. Different risk groups were classified using Durie and Salmon staging system (DS) (22), ISS (1), or R-ISS (2). Responses were assessed according to IMWG 2016 consensus criteria (23). Best response was defined as the maximal response during treatment. In treatment decision making, BD regimen has become a backbone to which several other agents have been integrated. BD regimen was administered every 4 weeks or 1 natural month, and 1.3 mg/m^2^ of bortezomib was administered subcutaneously weekly on days 1, 8, 15, and 22 with weekly dexamethasone 20 mg/m^2^ on the day of and the day after bortezomib administration. The third agent was added or omitted under certain conditions. Specifically, for patients with good tolerability to BD regimen, specific indications for initiation of the other therapy for MM existed. For patients with renal impairment and/or amyloidosis attributable to MM, cyclophosphamide was in combination with BD regimens using a 300 mg/m^2^ dosing on days 1, 8, 15, and 22 per cycle. Patients with extramedullary involvement received 40 mg of pegylated liposomal doxorubicin (in combination with BD backbone) on days 1 of each cycle. Following the BD regimen induction, stem cell harvest is all recommended for patients who met eligibility criteria (i.e., ≤65 years and ≥very good partial response (VGPR) in first remission). Upfront autologous stem cell transplantation was performed for patients from whose consent was obtained. For transplant ineligible patients, maintenance therapy was given after nine cycles of induction or treatment response was stable for three cycles of VGPR or above. All patients were given routine maintenance for at least 2 years with either lenalidomide or bortezomib, given both patients’ intent and risk stratification. This study was approved by our hospital’s institutional review board/research ethics board. All subjects provided written informed consent authorizing the use of their data for research purposes.

### Imaging Studying

FDG-PET/CT scan was performed according to the European Association of Nuclear Medicine guidelines version 1.0 and, from February 2015, version 2.0. Steps of PET/CT scan were reported previously (24). Simple, circular regions-of-interest (ROIs) were drawn by hand on axial, coronal, or sagittal co-registered PET/CT slices. SUVmax was obtained and corrected for body weight using the standard formula: mean ROI activity (MBq/ml)/[injected dose (MBq)/body weight (kg)] (25). ROIs were placed manually over all lesions, and the SUVmax was recorded for every lesion. Also, the highest SUVmax for every PET/CT scan was recorded, and these lesions were identified as indicator lesions. In an effort to standardize the interpretation of the baseline PET/CT scans, the criteria in three previous papers of the groups of Bologna and Udine were adopted in this study (6, 10, 26). Briefly, positive PET/CT findings were defined either by the presence of focal areas of increased tracer uptake within the bones, with or without any underlying lesions identified on CT presented on at least two consecutive slices, or by a SUVmax ≥ 2.5 within the osteolytic CT areas > 1.0 cm in size or a SUVmax ≥ 1.5 within the osteolytic CT areas ≤ 1.0 cm. The number, size, and location of hypermetabolic focal lesions (FLs) were recorded, and FDG-avid tissue that was not contiguous to bone and arose in soft tissue according to CT examination was defined as extramedullary disease (EMD) tissue. SUVmax > 4.2 was continued to be considered an unfavorable cutoff value for therapeutic response and survival in this study, which has been confirmed in previous studies (6, 10, 26).

For PET/CT is subject to the constraints of interobserver reproducibility and an imperfect systemic description when using only SUVmax (27), the study compared the predictive ability of SUVmax and the ratios between SUVmax of the tumor lesions to liver (rPET) (27).

### Laboratory Investigations

Bregs were characterized as CD19^+^CD24^hi^CD38^hi^ in BM samples from NDMM patients by FCM as previously described (17, 18). Heparinized BM was obtained from NDMM patients prior to treatment. Briefly, BM mononuclear cells (BMMNCs) were isolated and washed twice in PBS. After discarding the supernatant, BMMNCs were incubated with antibodies against CD38 (PE-cy7), CD19 (FITC), and CD24 (APC) (BioLegend) for 15 min. Excess (unbound) antibodies were removed by washing with PBS, and cells were resuspended in 0.2 ml PBS for FCM detection (Beckman).

### Statistical Analysis

Distributions of PFS and overall survival (OS) were calculated using the Kaplan-Meier method, and differences among survival curves were analyzed by the log-rank test. PFS was defined as the time from diagnosis to progression or death from any cause. OS was defined as the time from diagnosis to death from any cause. Significant risk factors for both PFS and OS that showed a P < 0.10 on univariate analysis were further tested in the multivariate Cox proportional hazards regression analysis. The cutoffs for SUVmax, rPET, and Bregs’ ratios were identified after applying sequential log-rank tests and selecting the most powerful values for discriminating the outcomes. The chi square test and the Fisher’s exact test were used to test for the independence of categories. Statistical significance was defined when P < 0.05. SPSS 25.0 software was used to process all collected data.

## Results

### Patient Characteristics

A cohort of 120 NDMM patients was enrolled with median follow-up of 28.20 months (range 7.00–46.93 months), and their baseline characteristics were shown in **Table 1**. A total of 12 (10.0%) deaths and 32 (26.7%) cases with disease progression occurred. The overall response rate (ORR: [complete response (CR) + VGPR + partial response (PR)]) was 88.4%, with 19 in 101 evaluable patients achieving CR.

**Table 1:** NDMM patients’ demographic and clinical characteristics.

	Overall	Patients with PET/CT scan			Patients with Bregs detection		
		SUVmax > 4.2 n/N (%)	SUVmax ≤ 4.2 n/N (%)	P	Bregs’ ratios < 10% n/N (%)	Bregs’ ratios ≥ 10% n/N (%)	P
Male	64/120 (53.3)	36/59 (61.0)	22/52 (42.3)	0.058	16/28 (57.1)	14/24 (58.3)	0.931
>65 years	38/120 (31.7)	16/59 (27.1)	18/52 (34.6)	0.416	8/28 (28.6)	10/24 (41.7)	0.388
M-component							
IgG	67/114 (58.8)	37/64 (57.8)	27/64 (42.2)		15/26 (57.7)	11/26 (42.3)	
IgA	25/114 (21.9)	10/22 (45.5)	12/22 (54.5)		3/7 (42.9)	4/7 (57.1)	
Light chain	16/114 (16.0)	6/14 (42.9)	8/14 (57.1)		5/11 (45.5)	6/11 (54.5)	
LDH > 220 IU/L	53/113 (46.9)	31/54 (57.4)	21/51 (41.2)	0.096	13/28 (46.4)	9/20 (45.0)	0.922
β2-microglobulin > 5.5 mg/L	38/111 (34.2)	17/53 (32.1)	18/49 (36.7)	0.679	13/26 (50.0)	7/23 (30.4)	0.245
DS, stage III	84/107 (78.5)	42/53 (79.2)	38/46 (82.6)	0.800	22/28 (78.6)	15/18 (83.3)	0.727
ISS, stage III	36/105 (35.2)	18/52 (34.6)	16/45 (35.6)	0.923	11/27 (40.7)	6/17 (35.3)	0.761
R-ISS, stage III	38/101 (37.6)	20/51 (39.2)	16/43 (37.2)	0.842	10/26 (38.5)	9/18 (50.0)	0.542
FISH at diagnosis in MM							
P53 deletion	10/90 (11.1)	10/46 (21.7)	0/37 (0.0)	0.002^#^	5/26 (19.2)	0/21 (0.0)	0.056
1q21 gain	40/90 (44.4)	20/46 (43.5)	18/37 (48.6)	0.664	11/26 (42.3)	10/21 (47.6)	0.776
IgH translocation	36/90 (40.0)	19/46 (41.3)	14/37 (37.8)	0.823	10/26 (38.5)	9/21 (42.9)	0.775
Karyotype abnormalities	8/84 (9.5)	4/42 (9.5)	4/37 (10.8)	0.850	2/25 (8.0)	4/19 (21.2)	0.378
First-line transplantation	25/117 (21.4)	16/58 (27.6)	7/50 (14.0)	0.102	7/27 (25.9)	5/23 (21.7)	0.754
Best response							
≥PR	90/101 (89.1)	43/47 (91.5)	40/47 (85.1)	0.523	20/26 (76.9)	18/18 (100.0)	0.028
<PR	11/101 (10.9)	4/47 (8.5)	7/47 (14.9)		6/26 (23.1)	0/18 (0.0)	

NDMM, newly diagnosed multiple myeloma; PET/CT, positron-emission tomography/computed tomography; Bregs, regulatory B cells; SUVmax, maximum standard uptake value; LDH, lactate dehydrogenase; DS, Durie and Salmon staging system; ISS, International Staging System; R-ISS, Revised International Staging System; FISH, fluorescence in situ hybridization; PR, partial response. ^#^P < 0.05, determined by the chi square test and the Fisher’s exact test.

One hundred and fourteen patients received PET/CT scan at the onset of disease and 84.3% presented positive lesions by PET/CT scan. Median SUVmax was 4.22 (interquartile range 3.17–6.00), while 53.2% patients were found with elevated SUVmax (SUVmax > 4.2) at onset (**Supplementary Table S1**). The rates of p53 deletion were higher in patients with SUVmax > 4.2 (P = 0.002), while distribution of other baseline characteristics was summarized (**Table 1**). More p53 deletion was also seen in patients with elevated rPET (defined as rPET > 1.46; **Supplementary Table S2**).

Furthermore, 52 patients’ BM samples were collected before receiving treatment to uncover the median ratios of BM-derived Bregs within CD19^+^ B cells as 7.5% (interquartile range 1.1%–27.2%). Cutoff of Bregs’ ratios of 10% was defined by sequential log-rank tests for discriminating therapeutic response and survival. Decreased Bregs’ ratios (defined as Bregs’ ratios < 10%) were conformed in 24 (46.2%) patients (**Supplementary Table S1**). A similar trend to higher ratios of p53 deletion was also observed in patients with Bregs’ ratios < 10% (P = 0.056) (**Table 1**).

### Patients With SUVmax > 4.2 or Bregs’ Ratios < 10% Had Lower Quality of Response to Treatment

For patients with Bregs’ ratios < 10%, 23.1% could not reach PR, while 100% reached beyond PR in the subgroup without Bregs’ ratios ≥ 10% (P = 0.028) (**Table 1**). A similar trend towards lower quality of best response occurred on patients with SUVmax > 4.2 (**Table 1**). However, the difference between the response of patients with rPET > 1.46 or rPET ≤ 1.46 was small (< PR 5/41 *vs*. 4/42; ≥ PR 36/41 *vs*. 38/42; P = 0.738) (**Supplementary Table S2**).

**Figure 1:**
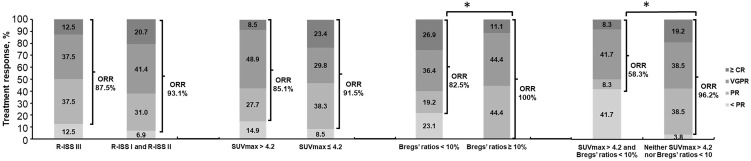
Overall response rates for all patients, by R-ISS III, SUVmax > 4.2, and Bregs’ ratios < 10% in NDMM. *P < 0.05, determined by the chi square test and the Fisher’s exact test. *NDMM*, newly diagnosed multiple myeloma; *ORR*, overall response rate (ORR = [complete response (CR) + very good partial response (VGPR) + partial response (PR)); *R-ISS*, Revised International Staging System; *SUVmax*, maximum standard uptake value; *Bregs*, regulatory B cells.

Additionally, as best response to first-line treatment, 6 cases (15.8%) with CR or stringent CR, 15 cases (39.5%) with VGPR, and 17 cases (44.7%) with or less than PR were observed among 38 evaluable patients who received both PET/CT scan and Bregs detection at diagnosis. The patients with both SUVmax > 4.2 and Bregs’ ratios < 10% presented the worst response with significant higher rates (41.7% *versus* 3.8%, P = 0.003) not reaching PR (**Figure 1**).

### SUVmax > 4.2, Bregs’ Ratios < 10%, as Well as R-ISS Ⅲ Were Independently Associated With Poorer Survival

**Figure 2:**
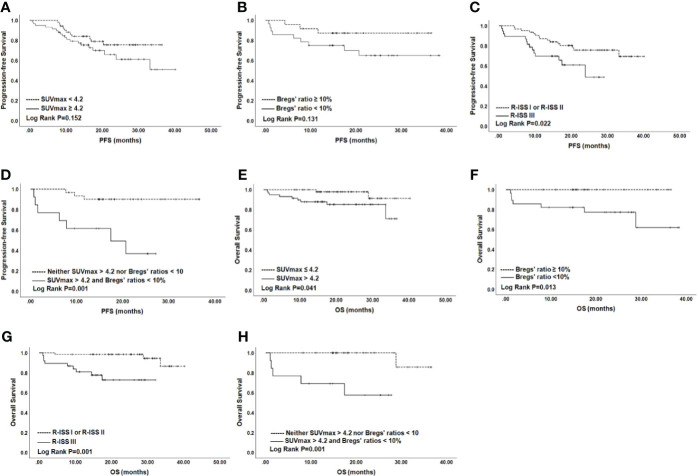
PFS and OS for NDMM patients by SUVmax **(A)**, Bregs **(B)**, and R-ISS **(C)**. OS according to SUVmax **(E)**, Bregs **(F)**, and R-ISS **(G)**. PFS **(D)** and OS **(H)** according to SUVmax >4.2 and Bregs’ ratios <10%. *NDMM*, newly diagnosed multiple myeloma; *PFS*, progression-free survival; *OS*, overall survival; *SUVmax*, maximum standard uptake value; *Bregs*, regulatory B cells; *R-ISS*, Revised International Staging System.

On univariate analysis for the whole cohort, R-ISS III, SUVmax > 4.2, and Bregs’ ratios < 10% predicted worse PFS, with hazard ratios (HR) (95% CI) of 2.35 (1.11–4.98), 2.05 (0.91–5.50), and 2.72 (0.86–8.59), respectively (P = 0.026, P = 0.074, and P = 0.089, respectively) (**Table 2**). To be specific, PFS for patients with SUVmax > 4.2 or Bregs’ ratios < 10% were significantly shorter than those observed for patients with lower SUVmax or higher Bregs’ ratios at the time of diagnosis (P = 0.152 and P = 0.131) (**Figure 2**). Similarly, patients with R-ISS III had median PFS (mPFS) of 23.83 months, in comparison with corresponding values of not reached (NR) (P = 0.001) for those who were with R-ISS I and R-ISS II (**Figure 2**). In particular, we compared the stratification ability of rPET and SUVmax for PFS, and elevated rPET (rPET > 1.46) did not perform better than SUVmax > 4.2 (**Supplementary Figure S2**). Age, sex, positive PET/CT findings, and FLs were found less predictive for PFS (**Supplementary Figure S2**). ISS III, p53 deletion, 1q21 gain, and IgH translocation were also found associated with worse PFS (**Table 2**). Female gender, age > 65 year, β2-microglobulin > 5.5 mg/L, with more than 3 FLs, and EMD did not show much significance on the univariate analysis. Thus, R-ISS III, SUVmax > 4.2, and Bregs’ ratios < 10% were included in the multivariate analysis, and it showed that the presence of R-ISS III, SUVmax > 4.2, and Bregs’ ratios < 10% were independent predictors of worse PFS (**Table 2**).

**Table 2:** Univariate and multivariate analysis of PFS and OS according to baseline risk variables for myeloma.

Univariate	95% CI					95% CI				
	HR	Lower		Upper	P	HR		Lower	Upper	P
	PFS					OS				
Sex (female)	1.16	0.57		2.33	0.684	1.29		0.41	4.06	0.665
Age > 65 years	1.03	0.49		2.17	0.943	0.42		0.09	1.90	0.258
LDH > 220 IU/L	1.60	0.78		4.27	0.201	2.30		0.69	7.64	0.176
β2-microglobulin > 5.5 mg/L	1.39	0.66		2.93	0.385	3.34		1.05	10.55	0.040
DS, stage III	1.67	0.64		4.36	0.298	1.58		0.35	7.21	0.557
ISS, stage III	1.88	0.91		3.91	0.090	8.37		2.17	32.28	0.002
R-ISS, stage III^§^	2.35	1.11		4.98	0.026	10.99		2.29	52.83	0.003
SUVmax > 4.2	2.05	0.91		5.50	0.074	4.32		0.93	20.06	0.062
>3 FLs^†^	0.99	0.62		1.59	0.974	0.90		0.43	1.87	0.772
EMD	1.22	0.37		4.05	0.740	4.94		0.86	19.26	0.121
Bregs’ ratios < 10%	2.72	0.86		8.59	0.089	2.88		0.85	8.29	0.192
FISH at diagnosis in MM										
P53 deletion	2.91	1.07		7.92	0.036	7.00		2.12	23.09	0.001
1q21 gain	2.37	0.99		5.65	0.052	2.24		0.65	7.66	0.200
IgH translocation	3.88	1.57		9.56	0.003	4.83		1.26	18.52	0.022
**Multivariate**	**95% CI**						**95% CI**			
	**HR**	**Lower**	**Upper**		**P**		**HR**	**Lower**	**Upper**	**P**	
	**PFS**					**OS**					
R-ISS, stage III^§^	5.45	1.03	28.81		0.046	R-ISS III§	8.67	1.82	41.20	0.007	
SUVmax > 4.2	5.13	2.49	12.31		0.039	SUVmax >4.2	4.41	0.94	20.55	0.059	
Bregs ratios < 10%	4.88	1.27	15.21		0.045						

PFS, progression-free survival; OS, overall survival; LDH, lactate dehydrogenase; DS, Durie and Salmon staging system; ISS, International Staging System; R-ISS, Revised International Staging System; SUVmax, maximum standard uptake value; FLs, focal lesions; EMD, extramedullary disease; Bregs, regulatory B cells; FISH fluorescence in situ hybridization.

^§^Referred to R-ISS I and R-ISS II.

^†^Referred to 1-3 FLs.

Univariate analysis for OS showed that SUVmax > 4.2, R-ISS III predicted worse OS with HR (95% CI) of 10.99 (2.29–52.83) and 4.32 (0.93–20.06) (P = 0.003 and P = 0.062). β2-microglobulin > 5.5 mg/L, lactate dehydrogenase (LDH) > 220 IU/L, ISS III, p53 deletion, and IgH translocation were also found associated with worse OS (**Table 2**). Similar to PFS, female gender, age >65 year, with more than 3 FLs, EMD, and rPET > 1.46 showed little impact on OS (**Supplementary Figure S2**). Thus, R-ISS III and SUVmax > 4.2 were included in the multivariate analysis and OS was negatively influenced by R-ISS III and SUVmax > 4.2 with HR (95% CI) of 9.97 (2.02–49.16) and 4.53 (0.97–21.11) (**Table 2**).

### Baseline PET/CT in Combination With Bregs as Prognosticators

The independent impact of elevated SUVmax and decreased Bregs’ ratios on PFS enabled us to stratify the NDMM patients into three groups, based on the number of risk factors (none of the two adverse factors, 26.8% of the patients; only one of two, 41.5%; two of the two adverse factors, 31.7%). As revealed by the results of Kaplan–Meier analysis and log-rank test, patients with both SUVmax > 4.2 and Bregs’ ratios < 10% experienced poorer PFS and OS than those with none factors (P = 0.001 and P = 0.001, respectively), although mPFS was not reached (**Figure 2**). Moreover, quality of response was worse for patients with both SUVmax > 4.2 and Bregs’ ratios < 10% (**Supplementary Figure S1**).

## Discussion

In this retrospective study of 120 NDMM patients who were evaluated at baseline using PET/CT scan and Bregs detection, we confirmed that imaging technique and TME-derived parameter have a good predictive value on the response of treatment, and both PET/CT > 4.2 and Bregs’ ratios < 10% can screen out a group of NDMM patients with poor survival. Furthermore, by combining SUVmax > 4.2 and Bregs’ ratios < 10%, a group of high-risk NDMM patients was stratified. To the best of our knowledge, this is the first report providing demonstration that PET/CT and Bregs predict response and survival in NDMM.

The predictive ability of SUVmax > 4.2 for poorer survival was confirmed again in this study, where the adverse influence of high SUVmax on the therapeutic response was explored at the same time. With the advent of PET/CT, bone destruction, tumor metabolism, and systemic tumor burden can be comprehensively evaluated; thus, PET/CT has likewise been combined with other parameters as prognosis prediction factors at baseline (5–7). Transplant-eligible NDMM patients with SUVmax > 4.2 were reported to have shorter PFS and OS (6). EMD and FLs > 3 were also adverse PFS and OS prognosticators for transplant-eligible NDMM patients (5, 6). For transplant-ineligible NDMM patients, PFS and OS were worse with the presence of SUVmax > 4.2, FLs > 3, and EMD (10). FLs > 3 and EMD on PET/CT were also correlated with significantly higher M protein and β2-microglobulin, more cytogenetic abnormalities in NDMM patients (28). In our study, more p53 deletion and elevated LDH were found in patients with SUVmax > 4.2, and we further confirmed the adverse impact of SUVmax > 4.2 on survival of NDMM patients. In the comparison between SUVmax and rPET, we found that the predictive ability of rPET was not stronger than that of SUVmax; it may be because the data of rPET had greater variability and the cutoff brought by the ratios did not have a better stratification effect. The adverse effect of SUVmax > 4.2 on the therapeutic response was explored in this study. As reported, achievement of deeper response within the first four cycles of treatment is an indicator for better survival (29, 30). In a more recent study, patients with a rapid PR or VGPR and gradually achieved CR were found with superior survival than those with early VGPR ≤ 3 months (31). In this study, 26.9% NDMM patients with Bregs ratios < 10% achieved best response of CR or stringent CR; we hypothesized this was because the prognosis of MM was affected by the response kinetics and duration of response in addition to the depth of response.

Positive correlation was found between the ratios of Bregs within CD 19^+^ B cells and NDMM patients’ outcomes in this study, and Bregs’ ratios < 10% was an appropriate cutoff for therapeutic response and survival in NDMM patients. The positive relationship between Bregs’ ratios and preserved B cells in NDMM was discovered in our previous study (17, 18). At time of relapse, CD19^+^ B cells, including Bregs, are too low to be detected (17). This is, to some extent, due to severe acquired immunodeficiency accompanying with a progressive depletion of lymphocytes, including CD19+ B cells, during relapse (32). The clinical behavior of MM is very heterogeneous; ^18^F-FDG PET/CT scan and Bregs detection can provide a more direct measure of tumor burden and TME and be exploited in an effort to identify newer prognostic factors, therefore improving prognosis.

R-ISS retained its prognostic significance in this study, and it was verified that R-ISS allowed the identification of three different groups of patients with clearly different outcomes. The comparison of the predictive value of R-ISS and other potential predictors in this study showed that R-ISS was an independent prognostic marker. SUVmax > 4.2 and Bregs’ ratios < 10% have shown the potential to screen a group of patients with poor PFS from NDMM patients. Moreover, patients with R-ISS III or patients with both SUVmax > 4.2 and Bregs’ ratios < 10% account for about 30% of the total NDMM population. PET/CT scan in combination with Bregs detection can complement R-ISS to achieve a good stratification of NDMM patients with poor prognosis. Since Bregs’ ratios < 10% was identified as an unfavorable prognostic variable for OS on the univariate analysis but not on the multivariate analysis, more patients are needed to be included and follow-up needs to continue to further consolidate the existing findings.

We acknowledge that there are several limitations to the current study. First, the study is retrospective and only a small number of patients are enrolled. Additionally, due to the short median follow-up time in this study, the predictive value of PET/CT scan and Bregs detection for the survival of NDMM patients cannot be fully revealed. Lastly, because PET/CT is not yet a standardized imaging tool in MM, the prognosis prediction value of SUVmax to response and survival of NDMM patients needs to be confirmed by more studies. Despite these limitations, the fact that PET/CT findings and frequency of Bregs within CD19^+^ B cells are well correlated with therapeutic response and survival is quite reliable.

In conclusion, adverse baseline PET/CT findings and low Bregs frequency were positively associated with poor therapeutic response and survival in NDMM patients. More attention is needed for a group of high-risk patients based on the definition of SUVmax > 4.2 and Bregs’ ratios < 10%, and risk-adapted treatment is required. On the basis of our results, integrating PET/CT scan and Bregs detection into the algorithm of NDMM staging may improve disease management and supplement risk stratification systems such as R-ISS. More studies are warranted to confirm our findings.

## Data Availability Statement

The raw data supporting the conclusions of this article will be made available by the authors, without undue reservation.

## Ethics Statement

The studies involving human participants were reviewed and approved by Institutional Review Board/Research Ethics Board of West China Hospital. The patients/participants provided their written informed consent to participate in this study. Written informed consent was obtained from the individual(s) for the publication of any potentially identifiable images or data included in this article.

## Author Contributions

JC: data analysis and interpretation and writing of manuscript. ZZ: detection of Bregs. JD: collection and assembly of data. WT: data analysis and interpretation. YL: collection and assembly of data. LZ: conceptualization and design, collection and assembly of data, supervision, and approval of final draft. LP: supervision and approval of final draft. TN: supervision and approval of final draft. All authors contributed to the article and approved the submitted version.

## Funding

This work was funded by support from the National Natural Science Foundation of China for general program (81770218).

## Conflict of Interest

The authors declare that the research was conducted in the absence of any commercial or financial relationships that could be construed as a potential conflict of interest.

## Publisher’s Note

All claims expressed in this article are solely those of the authors and do not necessarily represent those of their affiliated organizations, or those of the publisher, the editors and the reviewers. Any product that may be evaluated in this article, or claim that may be made by its manufacturer, is not guaranteed or endorsed by the publisher.
