# Supplementary material for: Predictive Values of PET/CT in Combination With Regulatory B Cells for Therapeutic Response and Survival in Contemporary Patients With Newly Diagnosed Multiple Myeloma
Source: Front Immunol. 2021 Aug 19;12:671904. doi: 10.3389/fimmu.2021.671904 (PMC8417409; doi:10.3389/fimmu.2021.671904)
Supplement: Supplementary file 1 [file DataSheet_1.docx]

**Supplementary materials**

Table S1 NDMM patients’ PET/CT and Bregs characteristics at baseline

| Characteristic | n/N (%) | Median (interquartile range) |
| --- | --- | --- |
| Patients with negative PET/CT | 19/114 (16.7) |  |
| Patients with positive PET/CT | 95/114 (84.3) |  |
| SUVmax |  | 4.22 (3.17-6.00) |
| rPET |  | 1.47 (1.00-2.08) |
| 1-3 FLs | 18/95 (18.9) |  |
| > 3 FLs or diffuse | 77/95 (81.1) |  |
| Patients with EMD | 10/114 (8.8) |  |
| Frequency of Bregs within CD19^+^ B cells, (%) |  | 7.45 (1.08-27.15) |
| Bregs’ ratios < 10% | 24/52 (46.2) |  |
| Bregs’ ratios ≥ 10% | 28/52 (53.8) |  |

*NDMM* newly diagnose multiple myeloma; *PET/CT* positron-emission tomography/computed tomography; *Bregs* regulatory B cells; *SUVmax* maximum standard uptake value; *rPET* the ratios of SUVmax in tumor lesions to SUVmax in the liver; *FLs* focal lesions; *EMD* extramedullary disease.

Table S2 NDMM patients’ PET/CT characteristics at baseline

|  | Patients with rPET > 1.46 n/N (%) | Patients with rPET ≤ 1.46 n/N (%) | P |
| --- | --- | --- | --- |
| Male | 32/47 (68.1) | 19/47 (40.4) | 0.013* |
| >65 years | 11/47 (23.4) | 17/47 (36.2) | 0.259 |
| M-component |  |  |  |
| IgG | 31/53 (58.5) | 22/53 (41.5) |  |
| IgA | 8/20 (40.0) | 12/20 (60.0) |  |
| Light chain | 4/12 (33.3) | 8/12 (66.7) |  |
| LDH > 220 IU/L | 24/45 (53.3) | 19/46 (41.3) | 0.297 |
| β2-microglobulin >5.5 mg/L | 14/43 (32.6) | 16/44 (36.4) | 0.822 |
| DS, Stage III | 34/43 (79.1) | 33/40 (82.5) | 0.784 |
| ISS, Stage III | 15/42 (35.7) | 14/39 (35.9) | 0.986 |
| R-ISS, Stage III | 19/43 (44.2) | 15/37 (40.5) | 0.822 |
| FISH at diagnosis in MM |  |  |  |
| P53 deletion | 9/39 (23.1) | 0/32 (0.0) | 0.003* |
| 1q21 gain | 16/39 (41.0) | 14/32 (43.8) | 0.817 |
| IgH translocation | 16/39 (41.0) | 13/32 (40.6) | 0.973 |
| Karyotype abnormalities | 3/36 (8.3) | 4/32 (12.5) | 0.699 |
| First-line transplantation | 12/45 (26.7) | 6/46 (13.0) | 0.121 |
| Best response |  |  |  |
| ≥ PR | 36/41 (87.8) | 38/42 (90.5) | 0.738 |
| < PR | 5/41 (12.2) | 4/42 (9.5) |  |

*NDMM* newly diagnose multiple myeloma; *PET/CT* positron-emission tomography/computed tomography; *rPET* the ratios of SUVmax in tumor lesions to SUVmax in the liver; *LDH* lactate dehydrogenase; *DS* Durie and Salmon staging system; *ISS* International Staging System; *R-ISS* Revised International Staging System; *FISH* fluorescence *in situ* hybridization; *PR* partial response. *P < 0.05, determined by the chi square test and the Fisher’s exact test.
