# Supplementary material for: Predictive Values of PET/CT in Combination With Regulatory B Cells for Therapeutic Response and Survival in Contemporary Patients With Newly Diagnosed Multiple Myeloma
Source: Front Immunol. 2021 Aug 19;12:671904. doi: 10.3389/fimmu.2021.671904 (PMC8417409; doi:10.3389/fimmu.2021.671904)
Supplement: Supplementary file 2 [file Presentation_1.pptx]

## Slide 1
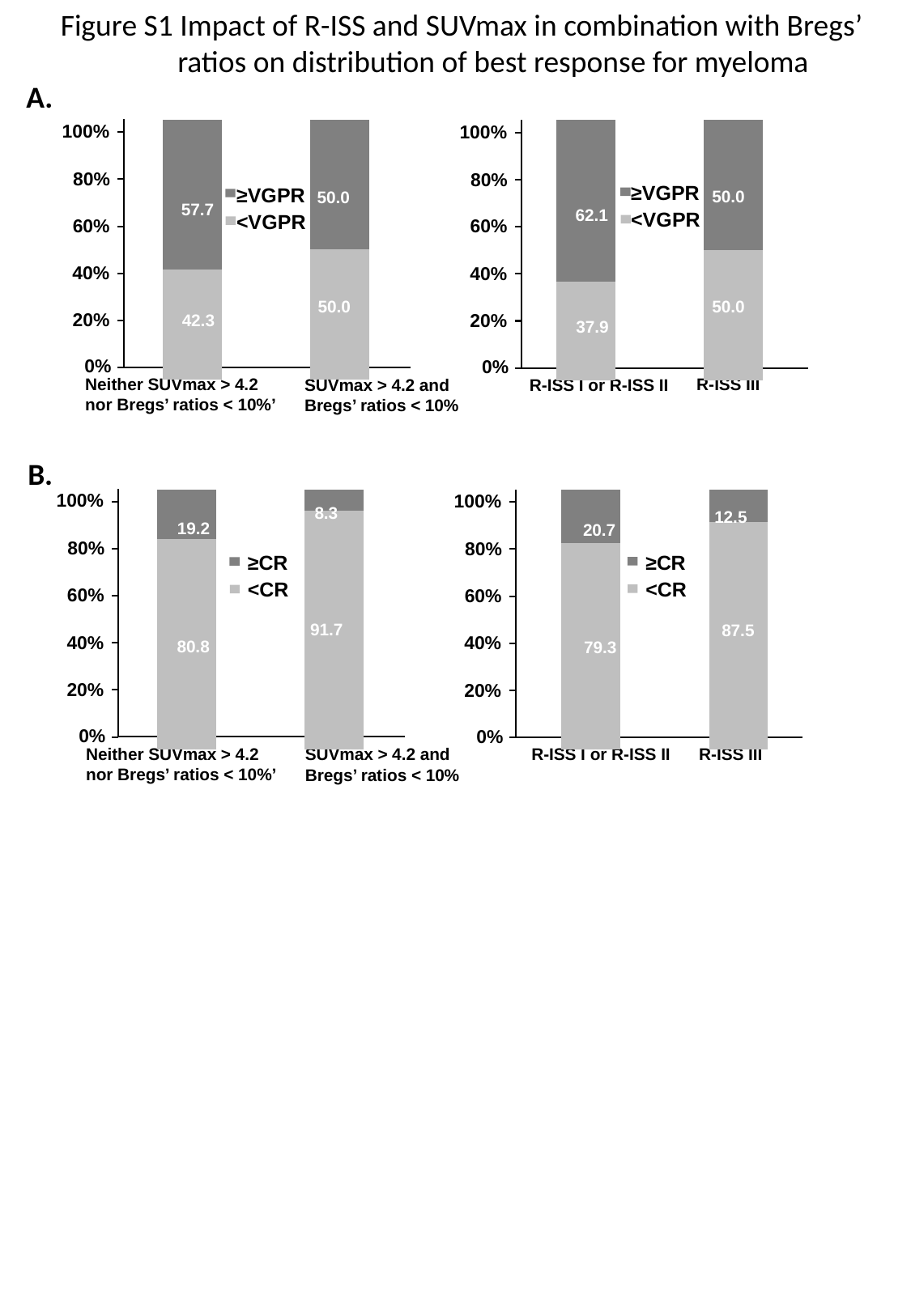

Figure S1 Impact of R-ISS and SUVmax in combination with Bregs’
 ratios on distribution of best response for myeloma
A.
### Chart
| Category | <VGPR | ≥VGPR |
|---|---|---|
| Score 0 or Score 1 | 11.0 | 15.0 |
| Score 2 | 6.0 | 6.0 |
### Chart
| Category | <VGPR | ≥VGPR |
|---|---|---|
| R-ISS Ⅰ or R-ISS Ⅱ | 22.0 | 36.0 |
| R-ISS　Ⅲ | 16.0 | 16.0 |
### Chart
| Category |
|---|100%
### Chart
| Category |
|---|80%
60%
40%
20%
0%
Neither SUVmax > 4.2
nor Bregs’ ratios < 10%’
SUVmax > 4.2 and
Bregs’ ratios < 10%
100%
80%
60%
40%
20%
0%
R-ISS III
R-ISS I or R-ISS II
≥VGPR
<VGPR
≥VGPR
<VGPR
50.0
50.0
57.7
62.1
50.0
50.0
42.3
37.9
B.
### Chart
| Category | <CR | ≥CR |
|---|---|---|
| Score 0 or Score 1 | 21.0 | 5.0 |
| Score 2 | 11.0 | 1.0 |
### Chart
| Category | <CR | ≥CR |
|---|---|---|
| R-ISS Ⅰ or R-ISS Ⅱ | 46.0 | 12.0 |
| R-ISS　Ⅲ | 28.0 | 4.0 |
### Chart
| Category |
|---|100%
### Chart
| Category |
|---|80%
60%
40%
20%
0%
Neither SUVmax > 4.2
nor Bregs’ ratios < 10%’
SUVmax > 4.2 and
Bregs’ ratios < 10%
100%
80%
60%
40%
20%
0%
R-ISS III
R-ISS I or R-ISS II
8.3
12.5
19.2
20.7
≥CR
<CR
≥CR
<CR
91.7
87.5
80.8
79.3

## Slide 2
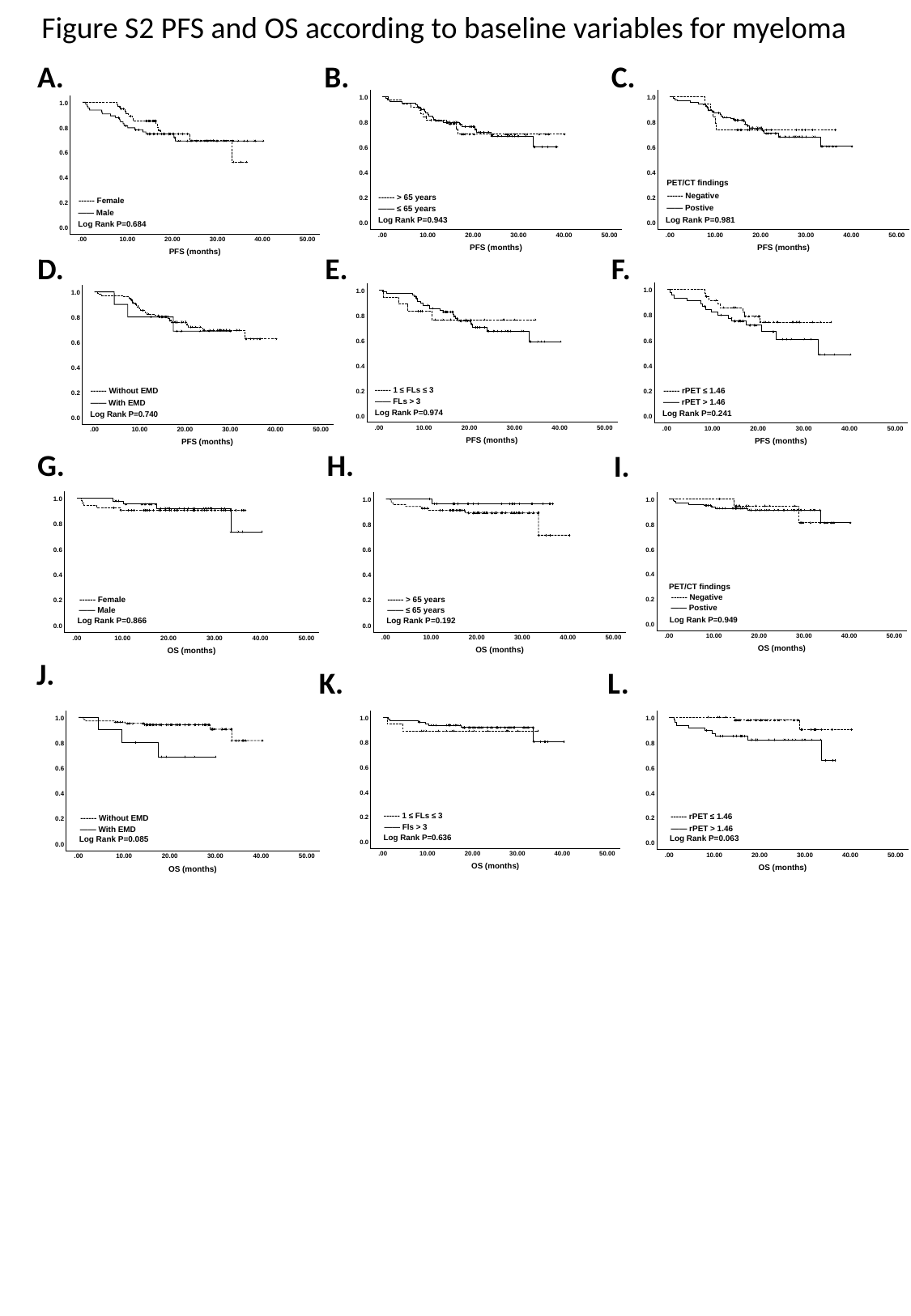

Figure S2 PFS and OS according to baseline variables for myeloma
A.
B.
C.
D.
E.
F.
G.
H.
I.
J.
K.
L.

## Slide 3
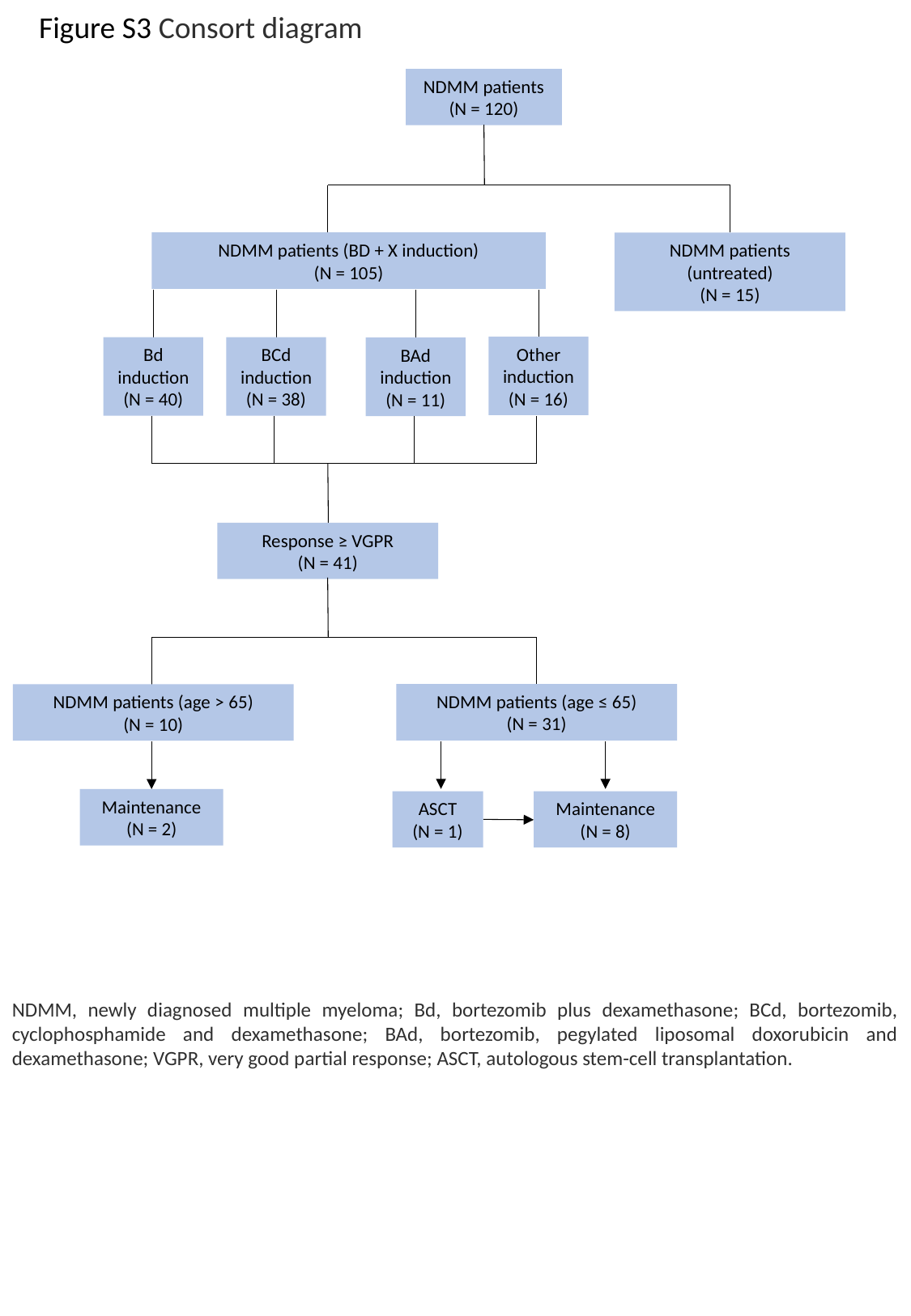

Figure S3 Consort diagram
NDMM patients
(N = 120)
NDMM patients (untreated)
(N = 15)
NDMM patients (BD + X induction)
(N = 105)
Other
induction
(N = 16)
Bd
induction
(N = 40)
BCd
induction
(N = 38)
BAd
induction
(N = 11)
Response ≥ VGPR
(N = 41)
NDMM patients (age ≤ 65)
(N = 31)
NDMM patients (age > 65)
(N = 10)
Maintenance
(N = 2)
ASCT
(N = 1)
Maintenance
(N = 8)
NDMM, newly diagnosed multiple myeloma; Bd, bortezomib plus dexamethasone; BCd, bortezomib, cyclophosphamide and dexamethasone; BAd, bortezomib, pegylated liposomal doxorubicin and dexamethasone; VGPR, very good partial response; ASCT, autologous stem-cell transplantation.
